# Toward a Better Understanding of Hip Adductor Function: Internal Rotation Capability Revealed by Anatomical and MRI Evaluation

**DOI:** 10.3390/jfmk10030354

**Published:** 2025-09-16

**Authors:** Kazuhiro Hirano, Kazuo Kinoshita, Atsushi Senoo, Masaru Watanabe

**Affiliations:** 1 Department of Molecular Physiology, The Jikei University School of Medicine, Tokyo 105-8461, Japan; 2 Department of Rehabilitation Medicine, The Jikei University Hospital, Tokyo 105-0003, Japan; kinop@jikei.ac.jp; 3 Department of Radiological Sciences, Graduate School of Human Health Sciences, Tokyo Metropolitan University, Tokyo 116-0012, Japan; senoo@tmu.ac.jp; 4 Department of Frontier Health Sciences, Graduate School of Human Health Sciences, Tokyo Metropolitan University, Tokyo 116-0012, Japan; masaru@tmu.ac.jp

**Keywords:** skeletal muscle, adductor muscle, pectineus, adductor longus, adductor brevis, functional anatomy, rotational function, T_2_ value

## Abstract

**Background**: At present, the rotational function of the hip adductor muscle group remains unclear. This study aimed to clarify the rotational function and stabilizing role of the pectineus, adductor longus, and adductor brevis (adductor muscle group) based on anatomical findings and T_2_ values (ms) obtained from magnetic resonance imaging (MRI). T_2_ values are prolonged in tissues with higher water content, and in skeletal muscle, it has been demonstrated that T_2_ values increase in proportion to exercise intensity. **Methods**: Using fixed specimens (*n* = 6, aged 61–96 years), we observed the three-dimensional arrangement of muscles in the neutral position of the hip joint and observed the extension or shortening of muscles associated with passive maximum internal and external rotation of the hip joint. In addition, we evaluated the activity of the adductor muscle group by T_2_ values (ms) from MRI pre- and post-internal rotation (forward step with the left leg) and pre- and post-external rotation (backward step with the left leg) movements of the right hip joint in a standing position (*n* = 8, healthy adult subjects, mean age 29.1 ± 5.3 years). **Results**: Regarding functional anatomy, the arrangement of the gluteus minimus and adductor muscle groups was almost parallel across the femoral neck. In the evaluation of adductor muscle group activity using MRI, the percent change in T_2_ values (%) of the pectineus was 6.38 ± 1.35 pre- and post-internal rotation and 1.35 ± 0.71 pre- and post-external rotation, whereas that of the adductor longus and brevis was 4.84 ± 1.31 pre- and post-internal rotation and 1.31 ± 0.68 pre- and post-external rotation. The percent change in T_2_ values pre- and post-internal rotation exercise was significantly greater than that pre- and post-external rotation exercise in the pectineus, adductor longus, and brevis muscles (*p* < 0.05). **Conclusions**: The adductor muscle groups are suggested to contribute to joint stability in the coronal plane and provide joint internal rotation in the standing position.

## 1. Introduction

The hip adductor muscle group consists of five muscles and is primarily responsible for hip adduction. Among them, the pectineus, adductor longus, and brevis not only have hip flexion in the neutral position of the hip joint, but can also switch to hip extension in the flexed position [1,2]. Currently, views on hip rotation in the hip adductor muscle group differ. No consensus is found in anatomy textbooks or previous studies regarding which muscles have internal or external rotation [1,2,3,4,5,6,7,8,9,10,11]. For example, anatomical textbooks on hip rotation reported inconsistent descriptions on internal rotation [3,4,5,6,7], external rotation [8,9], and internal and external rotation [10]. Furthermore, from a functional anatomical perspective, some reports suggested that the hip adductor muscle group is responsible for internal hip rotation [1,2], whereas others suggested that it is responsible for external hip rotation [11]. Consequently, hip rotation in the hip adductor muscle group remains unclear.

Human anatomy is an effective means of understanding functional anatomy (i.e., how skeletal muscles contribute to joint movement). The advantage is that the degree of muscle shortening and lengthening with changes in joint angles can be observed in three dimensions, inferring muscle function. Previous studies attempted to elucidate muscle function from an anatomical viewpoint using this advantage [12,13]. However, confirming whether the functional anatomical findings obtained during autopsy are the same in living humans is necessary.

To evaluate the activity state of skeletal muscles during human movement, a method using transverse relaxation time (T_2_ value) measured using magnetic resonance imaging (MRI) was previously reported [14,15,16,17,18,19,20]. Studies showed that the rate of increase in T_2_ values was proportional to exercise intensity [15], that a positive correlation between exercise load and the rate of increase in T_2_ values existed [16], that the more free water was released, the greater the muscle contraction [17], and that the increase in T_2_ values correlated with the amount of muscle discharge during exercise [18]. These previous studies showed that MRI T_2_ values could be used to quantitatively assess skeletal muscle activity [15,16,17,18,19,20]. The advantages of MRI for evaluating skeletal muscle function include the following: (1) it is painless and noninvasive, (2) it can evaluate not only superficial muscles but also deep and pelvic muscles, and (3) it can evaluate many muscles simultaneously in any cross-section. These advantages suggest that MRI is suitable for evaluating the state of activity of the adductor muscles, which are composed of several muscles present from the superficial to the deepest layers of the thigh. Nevertheless, T_2_ values are influenced by several factors, such as muscle water content, fiber type composition, and exercise intensity. Moreover, potential differences may exist between healthy individuals and athletic populations due to training-induced adaptations in muscle morphology and metabolism. Although the present study involved only healthy adults, these considerations should be taken into account when interpreting T_2_-based assessments.

During gait, pelvic rotation accompanied by hip internal rotation plays a crucial role in swinging the lower limb forward. Hip internal rotation contributes to walking efficiency, enabling smooth and energy-efficient locomotion. It is also indispensable in sports movements, such as kicking in soccer and rugby or rapid changes in direction. Therefore, clarifying the anatomical functions and muscle activity characteristics of the muscles contributing to hip internal rotation is important for understanding walking efficiency, developing clinical rehabilitation strategies, and enhancing sports performance.

Detailed reports exist on the morphology and function of the adductor magnus [21,22]; however, no detailed reports on the morphology or rotational function of the pectineus, adductor longus, or brevis (adductor muscle group) have been published. This study aimed to clarify the function of the adductor muscle group through anatomical findings and evaluate skeletal muscle activity using MRI.

## 2. Materials and Methods

### 2.1. Functional Anatomy—Methods

The subjects were six fixed specimens (eight limbs, four males, two females, aged 61–96 years) with no obvious deformities or injuries to the hip joints, as determined by visual inspection. Specimens were prepared by longitudinal transection of the pubic symphysis and sacrum, transection of the lower limb from the knee joint, removal of the epidermis, connective tissue, vascular system, and some joint bursa, and exposure of the adductor muscle. The prepared specimens were used to observe the three-dimensional arrangement of muscles in the neutral position of the hip joint and stretching or shortening of muscles associated with passive maximal internal and external rotation movements of the hip joint.

### 2.2. Evaluation of Skeletal Muscle Activity by MRI—Methods

**Figure 1:**
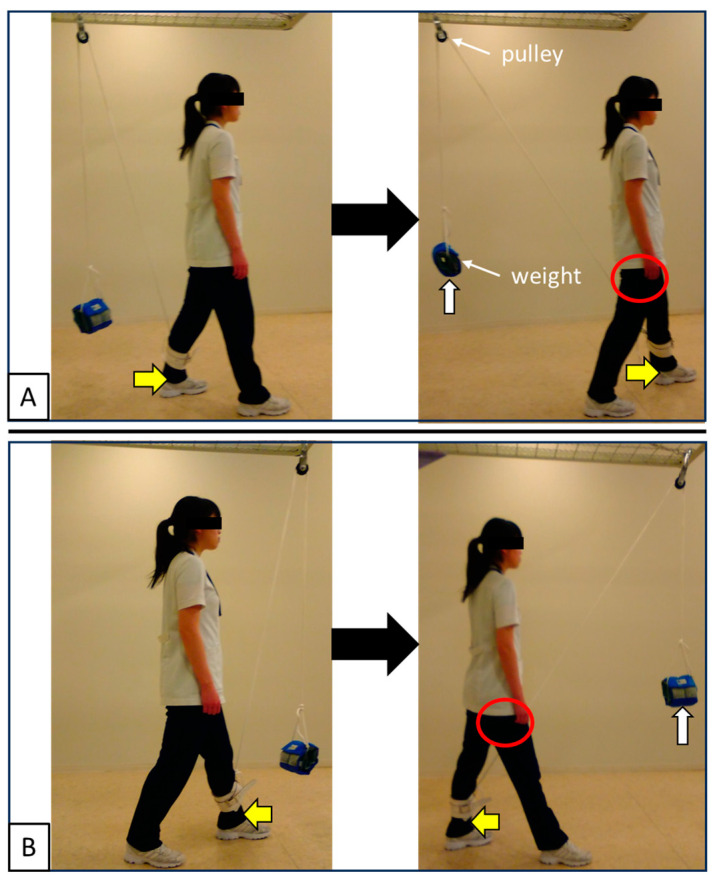
Exercise task. (**A**) Internal rotation of the right hip joint (forward step with the left leg). (**B**) External rotation of the right hip joint (backward step with the left leg). A weight approximately 10% of each subject’s body weight is loaded. The subjects performed the exercise while being aware of the rotation of their pelvis, and when returning the leg that had stepped, they did so with the weight of a weight while being as relaxed as possible. The red circle indicates the right hip joint, the yellow arrow indicates the step direction of the lower limb, and the white arrow indicates the direction in which the weight is lifted by the lower limb step. The models gave informed consent for publication in the paper.

Eight healthy adult volunteers (5 males and 3 females; mean age 29.1 ± 5.3 years, mean height 168.7 ± 6.4 cm, mean weight 58.4 ± 9.1 kg) participated in this study. Participants were recruited through convenience sampling from the university community. The inclusion criteria were age 20–40 years, no history of orthopedic disease in the lower limbs, and no regular exercise habits. Exclusion criteria included any contraindication to MRI (e.g., presence of metal implants, claustrophobia, or pregnancy). The number of participants was determined with reference to previous MRI-based studies of skeletal muscle activity, which typically included 5–7 subjects [16,18]. Accordingly, eight participants were recruited in the present study. Although the sample size was relatively small, it was considered sufficient for an exploratory investigation aimed at clarifying the rotational function of the adductor muscle group. The exercise task was to load approximately 10% of each participant’s body weight and perform internal (forward step with the left leg) and external rotation (backward step with the left leg) of the right hip joint in a standing position (Figure 1). The exercise was performed using a weight-pulley system mounted on an overhead frame. In the starting position, the pulley was adjusted so that the weight was positioned approximately 20 cm lateral and 20 cm anterior or posterior relative to the left lateral malleolus. The rope length was set so that, in the starting position, the weight was suspended at a height of approximately 30 cm above the floor. The rope was connected to an ankle cuff worn on the participant’s left ankle, and the weight position was kept identical across all participants to ensure reproducibility. The participants performed the exercise while being aware of the rotation of their pelvis. When returning the leg that had stepped forward, they did so with a weight while being as relaxed as possible. A metronome was used during the exercise, and internal or external rotation movements of 1 time/3 s were randomly performed for 3 min each. The step length and width were determined at the discretion of the subject.

**Figure 2:**
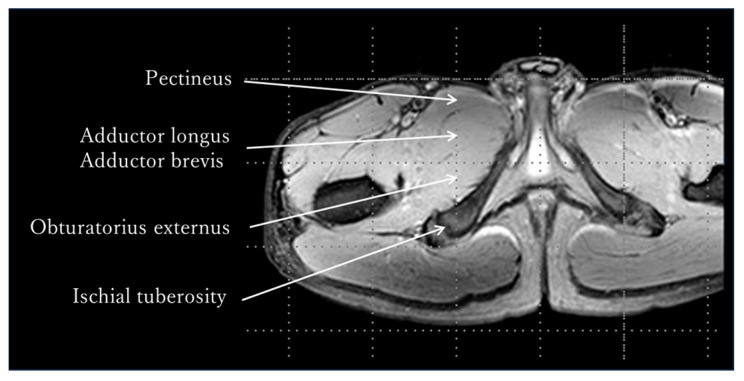
T_2_-weighted image. The adductor muscle group was identified in a transverse section at the tip of the ischial tuberosity, and the T_2_-weighted image was taken pre- and post-exercise using a Philips MRI system (Achieva 3.0T).

**Figure 3:**
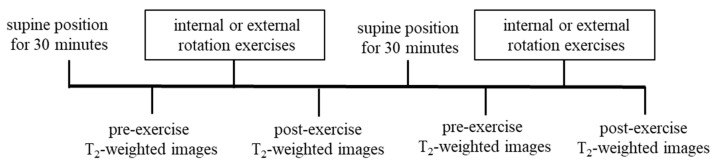
MRI measurement procedure. Internal or external rotation movements were performed randomly. A 30-minute rest period in the supine position was allowed between each movement.

**Figure 4:**
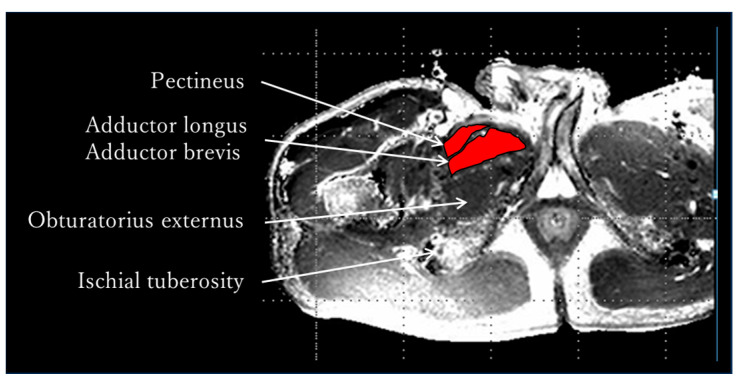
T_2_ Map. A T_2_ map was created from the captured images, and regions of interest (ROIs) were set in the pectineus, adductor longus and brevis. T_2_ values were measured using image analysis software MRIcro. The adductor longus and brevis were measured in the same ROI because it was sometimes difficult to clearly distinguish them on the images. Additionally, the ROI was set to avoid artifacts (longitudinal artefacts are visible on the lateral side of the adductor muscle group).

The MRI measurement procedure was as follows: After resting in supine position for 30 min, the adductor muscle group was identified in a transverse section at the tip of the ischial tuberosity using an MRI system (Achieva 3.0T, Philips Medical Systems, Amsterdam, The Netherlands), and pre-exercise T_2_-weighted images were captured using Multishot’s GraSE method (Figure 2). The limb position for imaging was supine on the imaging table, and the lower limbs were fixed with sandbags so that both hip joints were in a neutral position of adduction and abduction and internal and external rotation. After capturing the pre-exercise images, either internal or external rotation exercises were performed randomly. The adductor muscle group was identified at the tip of the ischial tuberosity as noted above, and post-exercise T_2_-weighted images were obtained immediately post each exercise. Additionally, a rest period of 30 min or more was provided between the internal and external rotation exercises (Figure 3). The sequence was as follows: matrix, 512 × 512; TR, 3900 ms, TE, 20 ms; slice, 1; slice thickness, 5 mm; echo, 5. The imaging times were 5 min and 28 s, respectively. A T_2_ map was created from the captured images, regions of interest (ROIs) were set in the pectineus, adductor longus, and brevis, and T_2_ values were measured using image analysis software MRIcro Version 1.9.1 (http://www.mricro.com; accessed on 13 September 2022) (Figure 4). The adductor longus and brevis were measured in the same ROI because it was sometimes difficult to clearly distinguish them in the images. The Shapiro–Wilk test and F-test were used to assess the normality and variance of the obtained T_2_ values between the pre- and post-exercise groups. The pre- and post-exercise T_2_ values, as well as their percent changes, were analyzed using a paired *t*-test to determine differences in adductor muscle group activity between internal and external rotation exercises. Furthermore, statistical analysis was performed on the effect size (Hedges’ g) and power (1 − β). The reliability of the T_2_ values was confirmed using the intraclass correlation coefficient (ICC). The ICC (1,1) values were measured twice by a physical therapist with 25 years of experience, whereas the ICC (2,1) values were measured by a physical therapist with 25 years of experience and a research assistant with no experience in skeletal muscle research. Modified R Commander 4.0.2 software (Windows version; https://home.hirosaki-u.ac.jp/pteiki/r/2modrdownload/ accessed on 10 December 2022) was used for the statistical analysis. Experimental data are expressed as mean ± SD.

The functional anatomy experiment was performed with the approval of the Ethics Committee of Jikei University School of Medicine (Approval No. 24-232, 6998). The MRI experiment was performed with the approval of the Research Safety and Ethics Committee of the Arakawa Campus, Tokyo Metropolitan University (Approval No. 12090). During their lifetimes, donors provided written informed consent to participate in the anatomical studies. In addition, an MRI experiment was conducted after obtaining written informed consent from all the subjects. Functional anatomy and MRI experiments were performed in accordance with the Declaration of Helsinki.

## 3. Results

### 3.1. Functional Anatomy—Results

**Figure 5:**
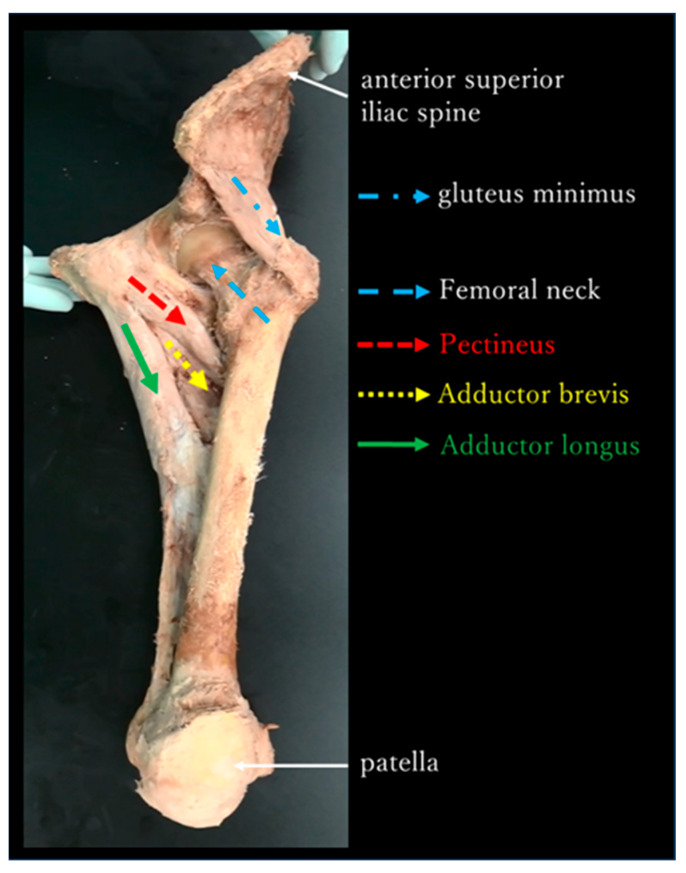
Front view of the left hip in neutral position. The arrangement of the adductor muscle group is almost parallel to the femoral neck and gluteus minimus in the frontal plane.

**Figure 6:**
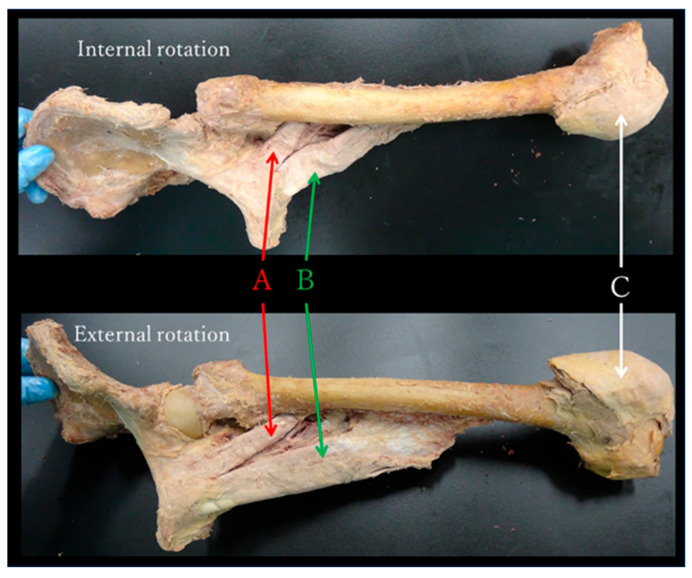
Passive movements of maximum internal and external rotation of the left hip joint. During passive movement, internal and external rotation of the hip joint shortened and stretched the adductor muscle group, respectively, in all specimens. (**A**) Pectineus, (**B**) Adductor longus, (**C**) Patella.

The adductor muscle group in the neutral position of the hip joint was almost parallel to the femoral neck and gluteus minimus in the frontal plane (Figure 5). During passive movement, internal and external rotations of the hip joint induced shortening and stretching of the adductor muscle group, respectively, in all specimens (Figure 6).

### 3.2. Evaluation of Skeletal Muscle Activity by MRI—Results

**Figure 7:**
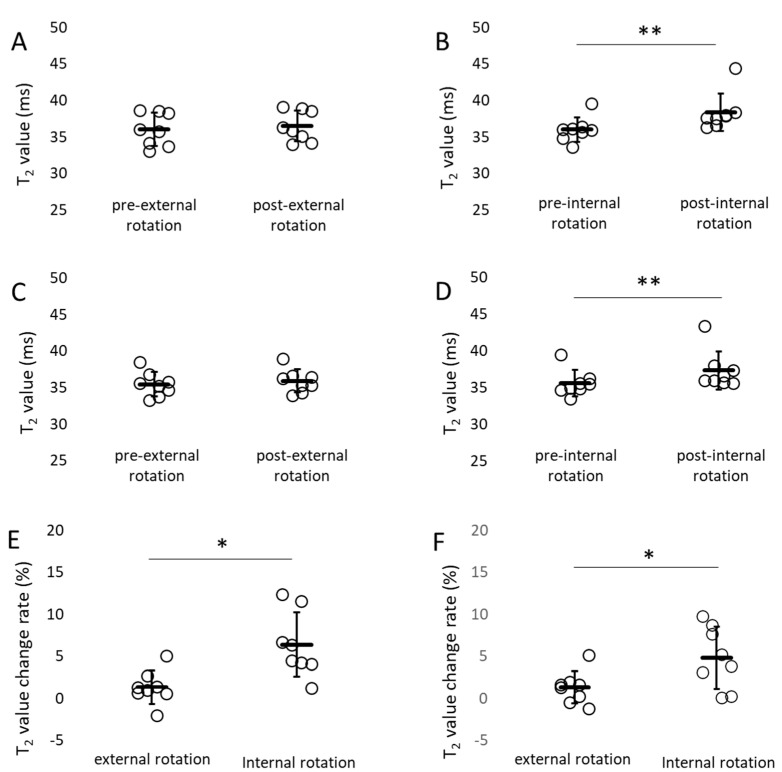
T_2_ value and percent change in T_2_ value. (**A**,**B**) T_2_ value of pectineus, (**C**,**D**) T_2_ value of adductor longus and brevis, (**E**) percent change in T_2_ value of pectineus, (**F**) percent change in T_2_ value of adductor longus and brevis. The T_2_ values pre- and post-exercise were not significantly different for external rotation exercises. The T_2_ values post internal rotation exercises were significantly prolonged compared with those pre internal rotation exercises. The percent change in T_2_ values pre- and post-internal rotation exercise was significantly higher than that pre- and post-external rotation exercise for pectineus, adductor longus, and brevis. mean ± SD (line and whiskers), ** *p* < 0.01. * *p* < 0.05.

Table 1 shows the normality and homogeneity of variances for T_2_ values. All *p*-values were greater than or equal to 0.05, confirming normal distribution and equal variance. The T_2_ values pre- and post-exercise were not significantly different for external rotation exercises. The T_2_ values post internal rotation exercises were significantly prolonged compared with those pre internal rotation exercises (Figure 7). The percent change in T_2_ values (%) was 6.38 ± 1.35 pre- and post-internal rotation and 1.35 ± 0.71 pre- and post-external rotation of the pectineus; and 4.84 ± 1.31 for internal rotation and 1.31 ± 0.68 for external rotation of the adductor longus and brevis. The percent change in T_2_ values pre- and post-internal rotation exercise was significantly higher than that pre- and post-external rotation exercise for the pectineus, adductor longus, and brevis muscles (Figure 7). Effect sizes and power pre- and post-internal rotation exercise results were all > 0.8, confirming large effect sizes and high power (Table 2). Significant differences were also observed pre- and post-internal rotation based on the 95% confidence interval (CI), *t*-value, and degrees of freedom from the *t*-test (Table 3). The ICC results were all > 0.8 for ICC (1,1) and > 0.75 for ICC (2,1). High intra- and inter-rater reliabilities were observed (Table 4).

## 4. Discussion

This study revealed the following.

- The adductor muscle group in the neutral position of the hip joint was almost parallel to the femoral neck and gluteus minimus in the frontal plane (Figure 5).
- During passive movement, the internal rotation of the hip joint shortened the adductor muscle group (Figure 6).
- The percent change in T_2_ values pre- and post-internal rotation exercise in the adductor muscle group was significantly higher than that pre- and post-external rotation exercise (Figure 7).

### 4.1. Functional Anatomy—Discussion

Previous studies on the functional anatomy of the hip joint reported that the gluteus medius (posterior fibres), especially the gluteus minimus, compresses the hip joint as a joint stabiliser [13,19]. However, because the hip joint is a ball-and-socket joint, it rotates if the gluteus minimus contracts. A muscle on the opposite side of the joint, parallel to the gluteus minimus, must be present to compress the hip joint and maintain stability. In this study, we found that the adductor muscle group in the neutral position of the hip joint was almost parallel to the femoral neck and gluteus minimus in the frontal plane (Figure 5). Therefore, the gluteus medius (posterior fibres), gluteus minimus, and adductor muscle groups are responsible for joint stability in the anterior plane by compressing the hip joint in a coordinated manner.

Previous studies on functional anatomy investigated multiple muscles or biarticular muscles simultaneously [12,13] and predicted muscle function from moment arms using muscle models [1,2]. However, in this study, we retained only three single joint muscles (the pectineus, adductor longus, and brevis), focused only on the rotational action of the hip joint, and observed changes in muscle length when the joints were actually moved. Using this method, we discovered that the adductor muscle group was shortened during internal rotation of the hip joint. This study is the first (to our knowledge) functional anatomical evaluation of hip rotation action of the adductor muscle group based on muscle length changes using cadavers. The present results demonstrate the that adductor muscle group was shortened and stretched when the hip joint was internally and externally rotated in all the specimens, suggesting that the adductor muscle group performed internal rotation of the hip joint.

### 4.2. Evaluation of Skeletal Muscle Activity by MRI—Discussion

To confirm whether the adductor muscle group acts on hip internal rotation in living human beings, as indicated by the present results of functional anatomy, we performed an activity evaluation using MRI. Several methods have been used to evaluate muscle activity. Surface electromyography can only be used on superficial muscles and the electrodes may be displaced by muscle contraction, whereas needle electromyography is an invasive method. Although ultrasound can assess real-time activity, probe pressure and angle influence the image. However, MRI is noninvasive and can evaluate multiple muscles simultaneously in any cross-section, making it a suitable evaluation device for assessing the activity state of the adductor muscle group, which consists of multiple muscles and is present from the superficial to deep layers of the thigh. The disadvantages of MRI include that it is performed in a confined space, measurements cannot be taken if metal is present in the body, and it is expensive, although these had little effect on the measurements in this study. The statistical analyses verified that assumptions of normality and homoscedasticity were met. Importantly, the comparisons of pre- vs. post-internal rotation exercise yielded large effect sizes (Hedges’ g = 0.9–1.3) with high statistical power (0.85–0.95). These results imply that the likelihood of Type II error is minimal, supporting the robustness of the observed effects even in the context of a relatively small sample size (Table 1, Table 2 and Table 3). The present results showed that the percent change in T_2_ value in the adductor muscle group pre- and post-internal rotation exercises was significantly higher than that pre- and post-external rotation exercises (Figure 7). In addition, T_2_ values pre- and post-exercise were not significantly different for external rotation exercises. However, the T_2_ values post internal rotation were significantly prolonged compared with those pre internal rotation exercises (Figure 7). Furthermore, the ICCs showed high intra- and inter-rater reliabilities, indicating that the measured T_2_ values were highly reliable (Table 4). These results indicate that the adductor muscle group undergoes strong contraction during hip internal rotation. Although functional anatomy was performed on only three muscles (pectineus, adductor longus, and brevis), the results showed that the adductor muscle group had a hip internal rotation function even in human movements in which all muscles were attached. To our knowledge, this study is the first to evaluate the rotational function of the adductor muscle group using MRI. Clarification of the internal rotational function during actual movements will provide useful knowledge in clinical situations.

When considering lower-limb function in clinical situations, it is important to understand the movements that involve standing, which is the closed kinetic chain (CKC). This is because the CKC and the open kinetic chain (OKC) may have different effects on the joint when skeletal muscles contract. Furthermore, we believe that movements are almost never performed solely by internal or external rotation of the hip joint. Therefore, a stepping movement imitating walking was selected as the exercise task in this study. Our present results show that the T_2_ values for the adductor muscle group in the hip internal rotation exercise were significantly prolonged compared with those in the hip external rotation exercise, indicating that the adductor muscle group was active during hip internal rotation exercises in the standing position. Murray et al. [23] used stroboscopic imaging to measure pelvic rotation angle during walking in healthy adults in 20 s–60 s and reported a value of 10.0 ± 3.5°. The distal influence of the centre during walking is significant, and a pelvic rotation of only 10° increases the stride length and contributes to gait efficiency. If no rotation of the pelvis (internal rotation of the hip joint) occurs when walking, the gait will be like a so-called ‘military march’ or the gait will be vaulting. The tensor fascia latae and anterior fibres of the gluteus medius and gluteus minimus contribute to internal hip rotation [2,12]. Our results strongly indicate that the adductor muscle group, together with these muscles, plays an important role in pelvic rotation (internal rotation of the hip joint).

This study has several limitations. First, the anatomical observations were based on only six cadaveric specimens, most of which were elderly, potentially limiting the generalizability of the findings to younger populations. Second, the MRI analysis was conducted with only eight young healthy participants, and the small sample size and narrow age range restrict the applicability of the results to broader populations. Furthermore, although T_2_ values in MRI can assess whether muscles are active, they cannot directly evaluate the torque exerted on the joint. Therefore, the extent to which muscle activity contributes to joint motion remains unclear. Future studies with larger and more diverse cohorts, combined with biomechanical approaches such as three-dimensional motion analysis or musculoskeletal modeling, will be important to clarify not only the quantitative contribution of the adductor muscle group to hip joint torque and functional movement but also how these findings relate to gait efficiency, hip pathology, and rehabilitation practice.

## 5. Conclusions

This study investigated the function of the adductor muscle group based on anatomical findings and skeletal muscle activity evaluated using MRI. As a result, anatomical findings suggest that the adductor muscle group is responsible for joint stability in the frontal plane, along with the gluteus medius (posterior part), and gluteus minimus. Muscle activity evaluation using MRI showed that the adductor muscle group has an internal rotation action during standing.

## Figures and Tables

**Table 1:** Normality and homogeneity of variances for T_2_ values.

	Shapiro-Wilk Test		F-Test	
	Pre- vs. Post-External Rotation Exercise	Pre- vs. Post-Internal Rotation Exercise	Pre- vs. Post-External Rotation Exercise	Pre- vs. Post-Internal Rotation Exercise
Pectineus	0.45	0.41	0.86	1
Adductor longus and brevis	0.36	0.75	0.29	0.35

**Table 2:** Effect size analysis and power for the paired *t*-test.

	Effect Size Analysis (Hedges’ g)		Power (1 − β)	
	Pre- vs. Post-External Rotation Exercise	Pre- vs. Post-Internal Rotation Exercise	Pre- vs. Post-External Rotation Exercise	Pre- vs. Post-Internal Rotation Exercise
Pectineus	0.59	1.40	0.39	0.97
Adductor longus and brevis	0.58	1.10	0.38	0.86

**Table 3:** 95% confidence interval (CI), *t*-value, and degrees of freedom for the *t*-test.

	95% CI		*t*-Test Statistics (*t*-Value and Degrees of Freedom)	
	Pre- vs. Post-External Rotation Exercise	Pre- vs. Post-Internal Rotation Exercise	Pre- vs. Post-External Rotation Exercise	Pre- vs. Post-Internal Rotation Exercise
Pectineus	−0.10~1.04	1.11~3.51	1.94 7	4.55 7
Adductor longus and brevis	−0.11~1.01	0.59~2.88	1.91 7	3.58 7

**Table 4:** Results of the intraclass correlation coefficient of T_2_ values. ICC (1,1) values were measured twice by a physical therapist with 25 years of experience, whereas ICC (2,1) values were measured by a physical therapist with 25 years of experience and a research assistant with no experience in skeletal muscle research.

**ICC (1,1)**	**Pre-External Rotation Exercise**	**Post-External Rotation Exercise**	**Pre-Internal Rotation Exercise**	**Post-Internal Rotation Exercise**
Pectineus	0.82	0.99	0.99	0.97
Adductor longus and brevis	0.95	0.92	0.91	0.93
**ICC (2,1)**	**Pre-External Rotation Exercise**	**Post-External Rotation Exercise**	**Pre-Internal Rotation Exercise**	**Post-Internal Rotation Exercise**
Pectineus	0.89	0.86	0.85	0.94
Adductor longus and brevis	0.75	0.94	0.75	0.93

## Data Availability

The original contributions presented in this study are included in the article. Further inquiries can be directed to the corresponding author(s).

## Abbreviations

The following abbreviations are used in this manuscript:

MRI	Magnetic Resonance Imaging
ROI	Region of Interest
ICC	Intraclass Correlation Coefficient
CKC	Closed Kinetic Chain
OKC	Open Kinetic Chain
